# Ex vivo–expanded allogeneic V**δ**2 T cells specifically reduce reservoirs of HIV-1 following latency reversal

**DOI:** 10.1172/jci.insight.198185

**Published:** 2026-02-10

**Authors:** Brendan T. Mann, Marta Sanz, Alisha Chitrakar, Kayley Langlands, Marc Siegel, Natalia Soriano-Sarabia

**Affiliations:** 1 Department of Microbiology, Immunology and Tropical Medicine, and; 2 Department of Medicine, The George Washington University, Washington, DC, USA.

**Keywords:** AIDS/HIV, Immunology, Immunotherapy

## Abstract

Latently infected cells persist in people living with HIV (PWH) despite suppressive antiretroviral therapy (ART) and evade immune clearance. “Shock and Kill” cure strategies are hampered by insufficient enhancement of targeted immune responses following latency reversal. We previously demonstrated that autologous Vδ2 T cells from PWH retain anti-HIV activity and can reduce CD4^+^ T cell reservoirs, although their use in cure approaches is limited due to their dual role as a viral reservoir. However, promising clinical data in oncology shows that their unique MHC-unrestricted antigen recognition affords potent on-target cytotoxicity in the absence of graft-versus-host disease when used as an allogeneic adoptive cell therapy modality. Here, we found expanded allogeneic Vδ2 T cells specifically eliminated HIV-infected CD4^+^ T cells and monocyte-derived macrophages (MDM), overcoming inherent resistance to killing by other cell types such as NK and CD8^+^ T cells. Notably, we demonstrated that allogeneic Vδ2 T cells recognized and eliminated the HIV-latent CD4^+^ T cell reservoir following latency reversal. Our study provides evidence for developing an allogeneic γδ T cell therapy for HIV cure and warrants preclinical investigation in combination approaches.

## Abstract 2

Allogeneic Vdelta2 T cells reduce the latent reservoir of people living with HIV

## Introduction

A pool of cells that contain latent HIV-1 provirus is not eliminated despite adequate suppression of viral replication with antiretroviral therapy (ART) (1). These latent cellular reservoirs represent the principal barrier to curing persistent HIV-1 infection (2). Resting memory CD4^+^ T cells are considered the main contributor to the latent reservoir; however, recent evidence suggests myeloid cells such as monocytes or tissue-resident macrophages also harbor infectious provirus (3, 4). One of the current strategies to target cellular reservoirs is known as the “Shock and Kill” approach. This strategy utilizes latency reversing agents (LRAs) to reactivate viral replication, leading to the induction of apoptosis and/or immune recognition of infected cells (5). Several clinical studies have demonstrated the ability of LRAs to reactivate viral transcription in vivo, but the overall size of the reservoir remained unchanged potentially due to inadequate HIV-specific immune responses (6, 7). Efforts to enhance reservoir clearance have mostly focused on boosting autologous HIV-specific CD8^+^ T cells through vaccination or ex vivo expansion (8, 9). These approaches face several key challenges that may limit their efficacy, including the high prevalence of escape mutations within the latent reservoir, functional exhaustion from chronic stimulation, and inefficient trafficking or retention within tissue reservoirs such as the lymph nodes (10–12). Furthermore, latently infected CD4^+^ T cells may avoid CD8^+^ T cell cytotoxicity through nef-mediated downregulation of MHC class I or additional uncharacterized mechanisms (13, 14). Similarly, infected macrophages may be intrinsically resistant to killing by both CD8^+^ T cells and NK cells (15, 16). This raises another substantial hurdle for clearing potential tissue sanctuaries for persistent HIV such as the gastrointestinal tract and central nervous system where non-CD4^+^ T cells contribute to the latent reservoir (17, 18). Therefore, these limitations necessitate the investigation of alternative strategies utilizing other effector cell subsets with known anti-HIV function such as γδ T cells.

The 2 major subsets of human γδ T cells, Vδ1 and Vδ2, exert potent cytotoxicity against malignant and infected cells through MHC-independent mechanisms (19, 20). The Vδ2 subset predominates in peripheral blood and specifically recognizes low–molecular weight pyrophosphate isoprenoid intermediates of the mevalonate and nonmevalonate biosynthesis pathways (21, 22). Expansion of Vδ2 T cells can be readily induced by the manipulation of the mevalonate pathway with FDA-approved drugs used for osteoporosis called aminobisphosphonates (N-BPs) (22, 23). These compounds induce accumulation of endogenous isopentenyl pyrophosphate, the specific TCR-activating ligand for Vδ2 T cells. Activated Vδ2 T cells display high expression of the innate receptor NKG2D and FcγRIIIa (CD16), which expand their cytotoxic potential to the recognition of stress-induced ligands and antibody-dependent cellular cytotoxicity (24, 25). We and others have described CD16 as a marker of cytotoxic populations in people with HIV (PWH) and people without HIV (PWOH) (26, 27). In addition, Vδ2 T cells can induce cell extrinsic apoptosis of target cells through expression of tumor-necrosis superfamily (TNFSF) ligands FasL and TRAIL (28, 29). Due to the MHC-independent nature of γδ T cell recognition, the use of allogeneic Vδ2 T cells presents an attractive alternative strategy to autologous cells that is emerging as safe and efficacious adoptive cell therapy for the treatment of solid tumors (30, 31).

Both γδ T subsets have demonstrated cytotoxicity against HIV-infected CD4^+^ T cells (32–36). Additionally, our group has demonstrated that autologous Vδ2 T cells can specifically target and kill CD4^+^ T cell reservoirs from virally suppressed PWH following latency reversal (37). A critical limitation that precludes the use autologous γδ T cells as part of HIV cure approaches is that some subsets are also infected by HIV-1 and can act as a cellular reservoir (35, 36, 38). We hypothesized that allogeneic Vδ2 T cells expanded from PWOH could specifically target and eliminate latent cellular reservoirs providing a stepping stone toward broadening current therapeutic options for HIV cure.

In this study, we demonstrate that expanded allogeneic Vδ2 T cells specifically eliminate both CD4^+^ T cells and monocyte-derived macrophages (MDM) actively replicating virus in a dose-dependent manner. Notably, MDM’s known resistance to NK and CD8 T cell killing (15, 16) was overcome by employing higher effector/target (E:T) ratios of Vδ2 T cells. Using the clinically relevant latency clearance assay (LCA) (37, 39), we further confirmed that expanded allogeneic Vδ2 T cells effectively reduced latently infected CD4^+^ T cells from ART-suppressed PWH following latency reversal, exhibiting similar efficacy to autologous Vδ2 T cells. Collectively, our findings provide compelling evidence supporting the use of allogeneic Vδ2 T cells as a promising immunotherapeutic strategy for curing persistent HIV-1 infection. Our study warrants further preclinical investigation to assess safety, optimize dosing and pave the way for clinical translation.

## Results

### Comparable cytotoxic phenotype between expanded Vδ2 T cells from PWOH and ART-suppressed PWH.

**Figure 1:**
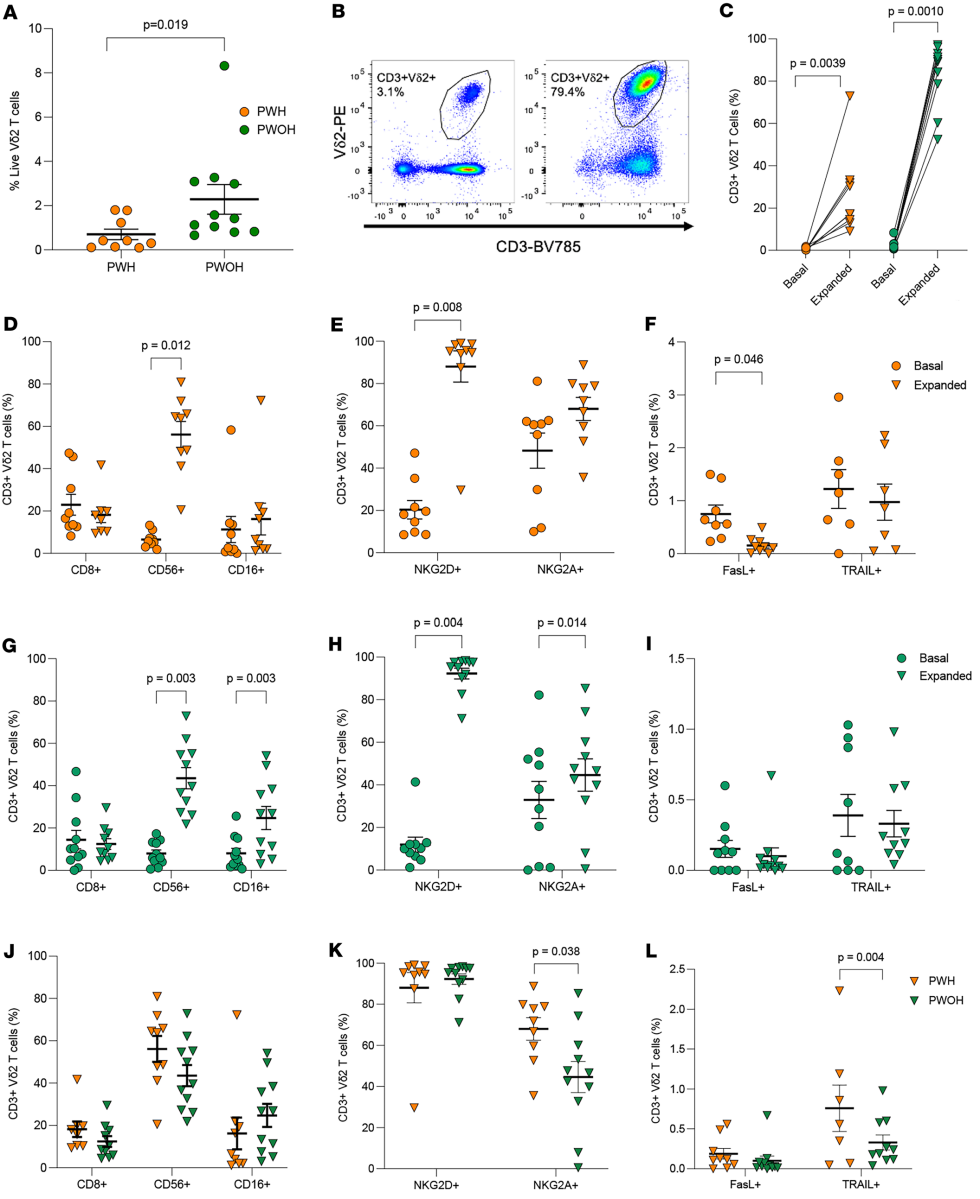
Comparison of pre- and post-Vδ2 T cell expansion between ART-suppressed PWH and PWOH. (**A**) Ex vivo (Basal) Vδ2 T cell frequencies. ART-suppressed PWH (*n* = 9), PWOH (*n* = 16). (**B** and C) Representative plots and quantification of Vδ2 T cells as a percentage of total live cells before (basal, circle) and after expansion (expanded, triangle) for 14 days. (**D**–**I**) Changes in cytotoxic markers in ART-suppressed PWH (**D**–**F**) and PWOH (**G**–**I**). (**J**–**L**) Comparison of expanded Vδ2 T cell phenotypes between ART-suppressed PWH (orange) and PWOH (green). Mann-Whitney *U* test (**A** and **J**–**L**). Wilcoxon matched pairs signed rank test (**C**–**I**).

HIV-1 infection results in the specific depletion of peripheral Vδ2 T cells through direct infection and gp120-mediated induction of apoptosis (35, 40). Although the frequency of Vδ2 T cells does not recover after durable viral suppression with ART, our work showed reconstitution of ex vivo N-BP–expanded Vδ2 T cells comparable with HIV-seronegative controls (27). For this study, PBMCs were isolated from a cohort of PWOH with a median age of 53 years old (IQR, 32–60) and even proportion (50%) of women and men. Additionally, PBMCs were isolated from a cohort of ART-suppressed PWH with a median age of 46 years old (IQR, 31–48) and slightly skewed toward male donors (62.5%) compared with female donors (37.5%). The median time since initiating ART was 8.1 years (IQR, 7.1–11.8) with a record of durable suppression for a median of 6.9 years (IQR, 3.8–8.0) (Table 1). Vδ2 T cells were specifically expanded with 3 μM alendronate (ALN) and 500 U/mL IL-2 for 14 days before being phenotyped by flow cytometry. Confirming our previous studies (27), Vδ2 T cells from PWH experienced a lower expansion compared with PWOH (mean 84.2 versus 26.5, or mean fold expansion of 59.1 versus 90.7, respectively. Mann-Whitney *U* test, *P* < 0.001). We focused on well-established markers associated with Vδ2 T cell cytotoxicity such as CD8, CD56, CD16, NK group 2 members NKG2D and NKG2A, and TNFSF ligands FasL and TRAIL (Supplemental Figure 1). In agreement with previous reports from our group and others (27, 37, 41), ART-suppressed PWH had a lower ex vivo frequency of Vδ2 T cells compared with controls (mean 0.7% versus 2.3%, *P* = 0.019; Figure 1A). Vδ2 T cells from both ART-suppressed PWH and PWOH expanded after 14 days in culture (Figure 1, B and C). As expected, the mean frequency of expanded Vδ2 T cells from PWH was lower than PWOH (mean 26.6% versus 82.3%, respectively; *P* < 0.001) and was not associated with biological sex or age of the donors (Supplemental Figure 2, A and B).

Expansion coincided with increased expression of CD56 and NKG2D (Figure 1, D and E) and decreased FasL expression (Figure 1F) in PWH, whereas in PWOH, we also observed an increase in CD16 and NKG2A (Figure 1, G and H) and no change in FasL or TRAIL (Figure 1I). CD16 was only upregulated in Vδ2 T cells expanded from PWOH (Figure 1, D and G).

The phenotype of expanded Vδ2 T cells was comparable between the 2 groups, with the only differences being a higher frequency of NKG2A and TRAIL-expressing cells in ART-suppressed PWH compared with PWOH (mean 68% versus 45%, *P* = 0.012; mean 0.76% versus 0.33%, *P* = 0.004; Figure 1, J–L).

Lastly, we did not find correlations between basal or expanded frequencies and phenotypes of our ART-suppressed donors and relevant clinical characteristics such as time since diagnosis, time on ART, time suppressed, or CD4 counts at the time of donation (not shown). These results confirm and expand our previous study that Vδ2 T cells can be robustly expanded from healthy PWOH that share phenotypic similarities with Vδ2 T cells expanded from ART-suppressed PWH (27).

### Ex vivo–expanded allogeneic Vδ2 T cells specifically kill both HIV-infected CD4^+^ T cells and MDMs.

**Figure 2:**
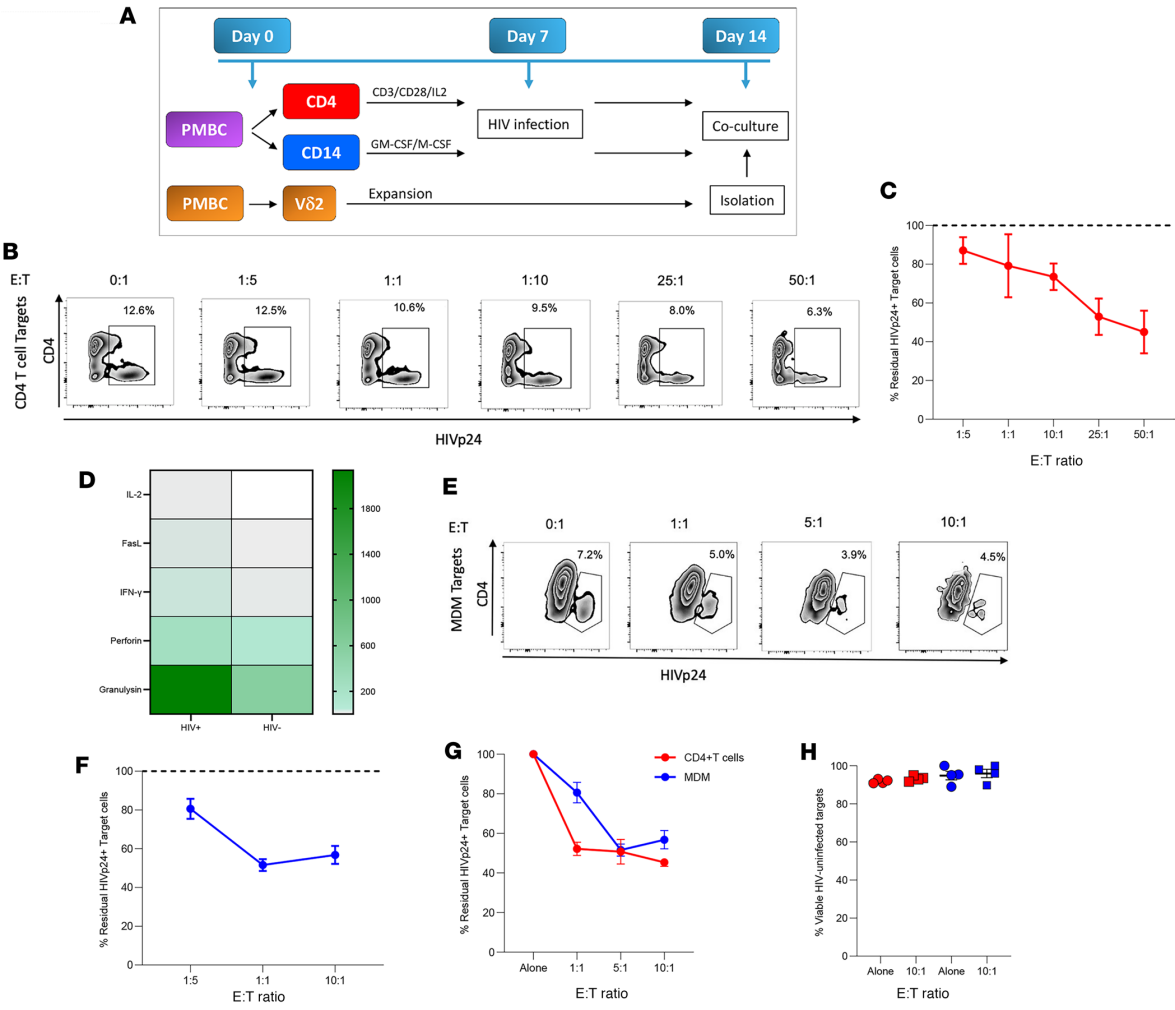
Allogeneic Vδ2 T cells kill HIV-infected CD4^+^ T cells and MDMs. (**A**) Schematic representation of CD4^+^ T cells and MDM target cell generation and Vδ2 T cell expansion. Target cells were infected with HIV_BAL_ prior to coculture with expanded allogeneic Vδ2 T cells. (**B** and **C**) Representative zebra plots and quantification of dose-dependent Vδ2 T cell-mediated killing of HIV-infected CD4^+^ T cells (*n* = 4). (**D**) Production of cytokine and cytotoxic soluble factors in supernatants from allogeneic Vδ2 T cells cocultured with either HIV-infected (HIV^+^) or uninfected (HIV^–^) CD4^+^ T cells at a 10:1 ratio and normalized to Vδ2 T cell monoculture controls. (**E** and **F**) Representative zebra plots and quantification of dose-dependent Vδ2 T cell-mediated killing of HIV-infected MDM cells (*n* = 4). (**G**) Head-to-head comparison of allogeneic Vδ2 T cell killing between HIV-infected CD4^+^ T cells (red) and MDMs (blue). (**H**) Viability of uninfected CD4^+^ T cell controls alone or cocultured with allogeneic Vδ2 T cells at 1 10:1 E:T ratio.

HIV-infected cells avoid recognition and killing by CD8^+^ T cells and NK cells through both viral and cell intrinsic mechanisms (13–16). Our previous studies show that autologous Vδ2 T cells from ART-suppressed PWH can inhibit active viral replication by specifically targeting infected CD4^+^ T cells (35, 37). Due to the MHC-independent nature of γδ T cell recognition, we hypothesized that allogeneic Vδ2 T cells would retain their ability to kill HIV-infected CD4^+^ T cells and macrophages. We modified a previously published method to generate CD4^+^ T cells and MDMs infected with HIV_BAL_ (15). Expanded allogeneic Vδ2 T cells were cocultured at different E:T ratios with either CD4^+^ T cells or MDMs for 18 hours, and intracellular HIVp24 production was analyzed by flow cytometry (Figure 2A). Coculture of infected CD4^+^ T cells with expanded allogeneic Vδ2 T cells from E:T ratios ranging from 1:5 through 50:1 resulted in a reduction in HIV_p24_^+^ target cells in a dose-dependent manner (Figure 2, B and C). A higher production of indicators of cell-killing IL-2, FasL, IFN-γ, perforin, and granulysin was observed in the supernatant of Vδ2 T cells cocultured with HIV-infected cells versus uninfected CD4^+^ T cells (Figure 2D).

Although we observed similar pattern of dose-dependent killing of HIV-infected MDMs, elimination plateaued between an E:T ratio of 5:1 and 10:1 (Figure 2, E and F). To test whether MDMs are resistant to Vδ2 T killing, we conducted a side-by side comparison between MDMs and CD4^+^ T cells from the same donors infected with HIV_BAL_. Comparison between CD4 and MDM killing revealed resistance to Vδ2 T cell killing at the 1:1 E:T ratio. However, this resistance was overcome when higher 5:1 and 10:1 ratios were applied (Figure 2G). Importantly, viability of uninfected CD4^+^ T cells was consistently high between cocultures with allogeneic Vδ2 T cells and monoculture controls (Figure 2H). These results demonstrate that ex vivo–expanded allogeneic Vδ2 T cells can specifically recognize and kill HIV-infected cells that may serve as latent reservoirs, including those resistant to killing by other effector cell types (15, 16).

### Ex vivo–expanded allogeneic Vδ2 T cells reduce the CD4^+^ T cell reservoir following latency reversal.

**Figure 3:**
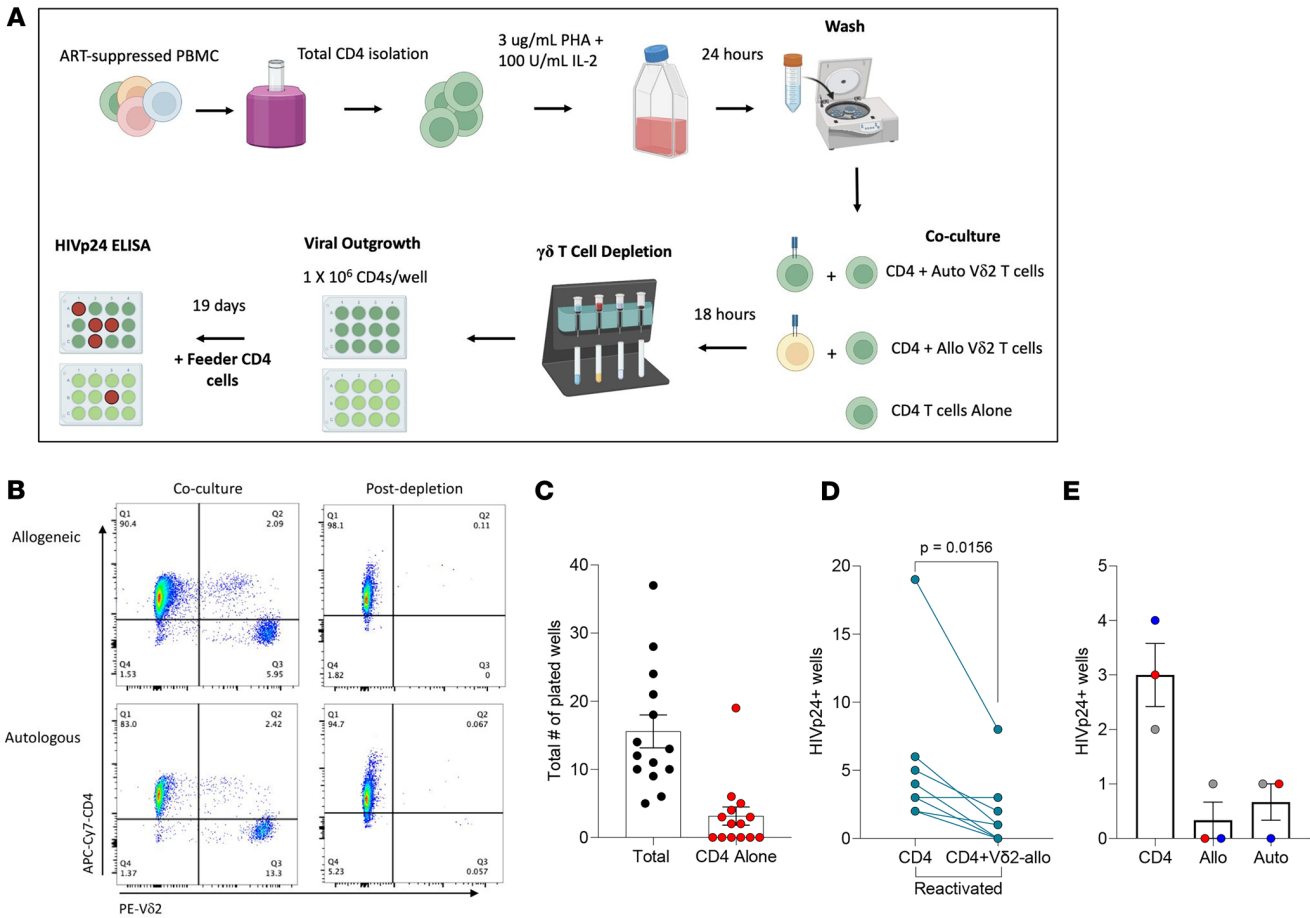
Allogeneic Vδ2 T cells eliminate reactivated latently infected CD4^+^ T cells. (**A**) Schematic representation of the latency clearance assay. (**B**) Purity of cocultures upon Vδ2 T cell depletion. (**C**) Total number of wells plated per assay (1 × 10^6^ CD4^+^ T cells/well) versus the total number of HIV_p24_^+^ wells from reactivated CD4^+^ T cell cultures at day 19 of outgrowth (*n* = 16). (**D**) Results of the LCA. Quantification of total HIV_p24_ positive wells in CD4^+^ T cells cultured alone compared with CD4^+^ T cells cocultured and depleted of allogeneic Vδ2 T cells following viral reactivation (*n* = 10). (**E**) Head-to-head comparison showing comparable reduction in HIV_p24_ wells between autologous versus allogeneic Vδ2 T cells (*n* = 3).

To assess whether allogeneic Vδ2 T cells can specifically eliminate latent reservoirs of HIV-1, we utilized an adapted version of the more clinically relevant LCA previously published by our group (37, 39). Total CD4^+^ T cells from ART-suppressed PWH were isolated, and viral transcription was induced by PHA and IL-2 for 18 hours. Following latency reversal, CD4^+^ T cells were cocultured with expanded allogeneic Vδ2 T cells for additional 18 hours (Figure 3A). Vδ2 T cells were then depleted from cultures (Figure 3B) to prevent nonspecific interactions with allogeneic feeder cells that are required to propagate viral outgrowth (37). Remaining CD4^+^ T cells were cultured for 19 days with allogeneic, activated CD4^+^ T cell feeders added on day 8. We detected positive viral outgrowth from CD4^+^ T cells cultured alone in 57.1% (8 of 14) of ART-suppressed PWH with a mean of 15.6 wells (1 × 10^6^ cells/well) plated and 3.1 positive wells (Figure 3C). Coculture with allogeneic Vδ2 T cells resulted in a reduction in the number of positive viral outgrowth wells when compared with CD4^+^ T cell monoculture controls (mean 1.9 versus 5.5 wells, respectively; *P* = 0.0156; Figure 3D), with 3 cases where no virus was recovered upon coculture. Lastly, we conducted a direct head-to-head comparison of autologous versus allogeneic Vδ2 T cells in 3 donors and found a comparable capacity to reduce the reactivated latent reservoir (Figure 3E). Together these findings demonstrate that allogeneic Vδ2 T cells can specifically recognize and eliminate the primary latent cellular reservoir of HIV-1 following viral reactivation.

## Discussion

In this study, we assessed the feasibility of using ex vivo–expanded allogeneic Vδ2 T cells as a potential therapeutic option to treat persistent HIV-1 infection. Confirming our own and others’ previous studies, ART-suppressed PWH displayed reduced capacity to expand in response to N-BP treatment compared with PWOH (27, 35). Both groups had a similar increase in the frequency of expanded Vδ2 T cells expressing CD56 and NKG2D. The expression of CD56 is associated with a subset of Vδ2 T cells with potent antitumor function mediated by high perforin and GzmB production (42, 43). Similarly, NKG2D is highly expressed on activated Vδ2 T cells and mediates the recognition of stress-induced ligands such as MICA, MICB, and ULBPs, which are upregulated on malignant and HIV-infected cells (24, 44, 45). Inhibiting NKG2D recognition of stress-induced ligands has been shown to reduce Vδ2 T cell cytotoxicity against HIV-infected CD4^+^ T cells and latently infected cell lines (35, 41). We found that only PWOH upregulated CD16 as a result of expansion. However, a direct comparison of expanded cells showed similar expression within both groups. The lack of change in CD16 expression contrasts our previous finding with a separate cohort of ART-suppressed PWH (27). CD16 expression marks a subset of Vδ2 T cells with increased cytotoxic rather than proliferative potential and shows extremely high variation within the general population (26). Therefore, it is possible that a greater sample size in this study would have identified similar differences to our previous work. Factors such as biological sex, age, and donor ethnicity influence Vδ2 T cell phenotypes and may also explain the differences between both study results (20, 27, 46). CD16 mediates Vδ2 T cell killing of HIV-infected cells through ADCC (47). Furthermore, we previously observed a strong negative correlation between CD16 expression on Vδ2 T cells and recovery of infectious virus from cocultures with reactivated CD4^+^ T cells, suggesting this population has enhanced anti-HIV capabilities (38). In this study, the cytotoxic phenotype of expanded Vδ2 T cells from PWOH displayed increased expression of several markers associated with anti-HIV effector functions observed in Vδ2 T cells from ART-suppressed individuals. These results show that allogeneic Vδ2 T cells display key functional characteristics critical for effective antiviral function and effective reservoir recognition.

Ex vivo–expanded allogeneic Vδ2 T cells were able to specifically target and kill both HIV-infected CD4^+^ T cell and MDMs in a dose-dependent manner. MDMs showed an initial resistance to killing at a lower E:T ratio of 1:1 compared with matching CD4^+^ T cell samples, and this resistance was overcome with the addition of more Vδ2 T cell effectors. It has been proposed that MDMs are generally more resistant to apoptosis compared with other cells types (48). However, previous reports suggest MDM resistance to CD8^+^ T cell and NK cell–mediated killing is due in part to prolonged engagement through the immune synapse (15, 49). The resistance to NK cells can be overcome through the engagement of additional receptors such as FasL or recognition of IgG-bound target through CD16, both of which are expressed on expanded Vδ2 T cells (16, 50). By contrast, Vδ2 T cells have been shown to eliminate influenza-infected MDMs through a combination of mechanisms involving NKG2D and FasL (51, 52). Our results provide further evidence that Vδ2 T cells are critical mediators of antiviral immunity toward virally infected myeloid cells. Importantly, we demonstrate that ex vivo–expanded allogeneic Vδ2 T cells can eliminate cell types known to harbor latent HIV-1 and are resistant to killing by other effector cell types. Determining the mechanism of Vδ2 T cell recognition of HIV-infected CD4^+^ T cells and MDMs is a critical next step to refine their therapeutic potential as a strategy for curing persistent HIV-1 infection.

We directly tested the ability of allogeneic Vδ2 T cells to eliminate reactivated latently infected CD4^+^ T cells from ART-suppressed PWH using the clinically relevant LCA (37, 53). A crucial step we previously optimized to this assay is the removal of Vδ2 T cells following coculture before viral outgrowth from CD4^+^ T cell targets. This removes the potential inhibitory effects of β chemokines produced by γδ T cells such as macrophage inflammatory protein-1α (MIP-1α), MIP-1β, and RANTES that inhibit infection through competitive binding to the CCR5 coreceptor (33, 54). We showed allogeneic Vδ2 T cells reduced the number of CD4^+^ T cells producing infectious virus following latency reversal. This reduction was comparable with autologous Vδ2 T cells in parallel comparisons. These data demonstrate that allogeneic Vδ2 T cells can specifically target latent reservoirs of HIV-1 within the context of the “Shock and Kill” approach. Due to the limited number of latently infected myeloid cells and lack of sufficient model systems, further confirmation of non-T cell reservoir clearance by Vδ2 T cells warrant further investigation (55). Recognition of viral reservoirs may entail a multilevel recognition that may differ between cells actively producing virus in the absence of antiretroviral versus cells undergoing latency reversal. If recognition is mediated by germline-encoded receptors such as NKG2D, then the choice of LRA has additional importance. Specifically, histone deacetylase inhibitors have been shown to augment the expression of NKG2D ligands following latency reversal (53, 56). Additionally, we have previously reported N-BPs, such as ALN, also possess the ability to reactivate viral transcription and activate autologous Vδ2 T cells (57). Combining ALN with the infusion of ex vivo–expanded allogeneic Vδ2 T cells could prove to be an advantageous strategy for HIV-1 cure (58). Allogeneic Vδ2 T cells have demonstrated promising safety and efficacy data in clinical studies for several solid tumors (30, 59). However, assessing safety of such an allogenic adoptive cell therapy within the context of persistent HIV-1 infection will be critical to translating this strategy for ART-suppressed PWOH who may possess a differentiated risk-benefit profile compared with oncology.

In summary, we have demonstrated that allogeneic Vδ2 T cells expanded from PWOH are highly cytotoxic effectors that retain key functional characteristics critical for effective reservoir clearance. Overall, our study provides preclinical foundation for the use of an off-the shelf immunotherapeutic strategy to eradicate latent reservoirs and move closer to a functional cure or sustained remission.

## Methods

### Sex as a biological variable.

Self-reported biological sex of study participants was included as a variable for the analysis of Vδ2 T cell expansion frequencies. Reported answers included male and female patients for both PWH and PWOH.

### Study participants.

PWH with over a year of viral suppression on ART were enrolled at the George Washington University Medical Faculty Associates, Washington DC, and the University of North Carolina, Chapel Hill. An additional 16 deidentified buffy coats from PWOH were purchased through the Gulf Coast Regional Blood Center (Houston, Texas, USA).

### Flow cytometry immunophenotyping and cell sorting.

Peripheral blood mononuclear cells (PBMCs) were isolated by Ficoll-gradient and viably stored in 20% DMSO in Liquid Nitrogen until further use. Cells were thawed and counted 3 times with a LUNA-FL cell counter (Logo Biosystems), stained for viability with fixable Zombie Aqua (BioLegend), and incubated with TruStain FcX (BioLegend) to prevent nonspecific binding. Following blocking, cells were stained with monoclonal antibodies (BioLegend) targeting CD3 (clone SK3), Vδ1 (clone REA117, Miltenyi), Vδ2 (clone B6), CD8 (clone SK1), CD56 (clone 5.1H11), CD16 (clone 3G8), NKG2D (clone 1D11), NKG2A (clone S19004C), FasL (clone NOK-1), and TRAIL (clone RIK-2) for 20 minutes in the dark at 4°C. Cells were then washed, fixed in 2% PFA, and acquired on a LSRFortessa X-20 (BD Biosciences) and analyzed using FlowJo software v10.10.0 (BD Biosciences). For LCAs, expanded Vδ2 T cells were sorted by FACS using a SH800 (Sony Biotechnology). Postsort purities of Vδ2 T cells were > 99.9% of live cells.

### Ex vivo expansion of Vδ2 T cells.

PBMCs were magnetically depleted of αβ T cells (StemCell Technologies) and cultured in complete RPMI (cRPMI) supplemented with 1% penicillin/streptomycin (Sigma-Aldrich) and 10% FBS (Atlas Biologics) plus 3 μM ALN (Sigma-Aldrich) and 500 U/mL IL-2 (Peprotech). Cells were cultured at a density of 1 × 10^6^/mL replenishing half of the media with 500 U/mL IL-2 every 2–3 days for a total of 14 days. At the end of the culture period, cells were harvested and immunophenotyping of expanded Vδ2 T cells conducted as described above. Expansions with Vδ2 T cells representing > 95% of total live cells were used in subsequent cytotoxic cocultures with HIV-infected target cells. For LCAs, highly purified Vδ2 T cells were isolated by FACS as described above.

### Generation of CD4^+^ T cells and MDM targets.

Target CD4^+^ T cells and MDMs were generated from PWOH, as previously described (16). Briefly, CD4^+^ T cells were isolated by negative magnetic selection (StemCell Technologies) and activated with 3 μg/mL of plate-bound αCD3 (clone OKT3), 5 μg/mL αCD28 (clone 28.2), and 100 U/mL of IL-2. Cells were cultured at a density of 1 × 10^6^/mL for 3 days, when the cells were washed and resuspended at 0.5 × 10^6^/mL in cRPMI and 100 U/mL IL-2 before being transferred to a culture flasks to allow for expansion for an additional 4 days.

In parallel, monocytes were isolated from PBMCs by positive magnetic selection for CD14^+^ cells (Miltenyi) and differentiated in the presence of cRPMI supplemented with 50 ng/mL M-CSF and 50 ng/mL GM-CSF (Peprotech). Cells were cultured at a density of 0.5 × 10^6^/mL in low attachment plates for a total of 7 days with media supplemented with 50 ng/mL M-CSF and 50 ng/mL GM-CSF. On day 7, maturation into macrophages was visually assessed for cell spreading on the surface of the plate.

### Infection of targets.

Freshly prepared target CD4^+^ T cells were washed twice and infected with strain HIV_BAL_ by spinoculation at 2,000*g* for 2 hours. Cells were washed twice to remove unbound virus and resuspended in cRPMI and 100 U/mL IL-2. Cells were seeded at a density of 1 × 10^6^/mL and cultured for 3 days to promote infection by cell-to-cell transmission. On day 7 of MDM maturation, half of the media was removed and replenished with fresh cRPMI supplemented with 50 ng/mL M-CSF and 50 ng/mL GM-CSF and HIV_BAL_ (NIH HIV Reagent Program) then incubated for 6 hours at 37°C. After incubation, virus-containing media were removed and replenished with fresh cRPMI and cytokines, and cells were cultured for 3 days.

### Cytotoxic coculture.

HIV-infected target cells were extensively washed and stained with 2.5 μM Cell Trace Violet (Thermo Fisher Scientific) in 1× PBS for 20 minutes at 37°C in the dark. The staining was then quenched with prewarmed cRPMI and incubated for an additional 5 minutes, washed, and resuspended in cRPMI at a concentration of 1 × 10^6^ cells/mL. CD4^+^ T cell targets were cocultured with expanded allogeneic Vδ2 T cells at varying E:T ratios ranging from 1:5 to 50:1 and plated in triplicate wells. MDMs were cocultured at E:T ratios ranging from 1:1 to 10:1. Target cells cultured alone were used as controls and all conditions were incubated at 37°C for 18 hours. Following coculture, cells were harvested, washed, and stained for viability with fixable Zombie NIR (BioLegend) before being incubated with TruStain FcX (BioLegend) to prevent nonspecific binding. Following blocking, conditions with CD4^+^ T cells were stained with monoclonal antibodies (BioLegend) targeting CD3 (clone SK3), Vδ2 (clone B6), and CD4 (clone SK3) whereas MDM cocultures were stained with CD3 (clone SK3), Vδ2 (clone B6), CD14 (clone HCD14), CD16 (clone 3G8), and CD4 (clone SK3) for 20 minutes in the dark at 4°C. Cells were then washed, fixed, and permeabilized with Cytofix/Cytoperm (BD Biosciences) for 20 minutes at 4°C. Following permeabilization, cells were washed in 1× Perm/Wash Buffer then stained with a monoclonal antibody specific for HIVp24 (clone KC57, Beckman Coulter) for 20 minutes at 4°C. Samples were acquired on a LSR-Fortessa X-20 (BD Biosciences) and analyzed using FlowJo software v10.10.0 (BD Biosciences).

### Cytotoxic coculture analyte detection.

Supernatant was harvested following 18 hours from 10:1 cocultures as well as CD4^+^ T cell alone and Vδ2 T cell monoculture controls then stored at –80°C. Supernatants were then gently thawed, and 25 μL were used to detect cytotoxic analytes and cytokines using the Legendplex Human CD8/NK Panel Detection kit (BioLegend). Samples and standards were plated in technical duplicates. All samples were acquired on an LSR-Fortessa X-20 (BD Biosciences) and analyzed using the Legendplex Data Analysis Software Suite v2025.05.01. Results are represented as the comparison between HIV-infected CD4 cocultures with uninfected CD4 cells.

### LCA.

An adapted version of the LCA was performed, as previously described by our group (37). Briefly, PBMCs from ART-suppressed PWH were thawed and rested overnight in 10 nM Abacavir and 0.5 μM Nelfinavir (NIH HIV Reagent Program) to prevent new rounds of infection. The cells were washed to remove antiretrovirals, then CD4^+^ T cells were isolated by negative magnetic selection (StemCell Technologies) and viral replication was reactivated for 18 hours using 3 μg/mL phytohemagglutinin (PHA) and 100 U/mL IL-2 (Peprotech). Following reactivation, CD4^+^ T cells were washed twice and cocultured with FACS-purified expanded Vδ2 T cells at a 1:5 E:T ratio or alone for additional 18 hours. Vδ2 T cells were depleted from cultures by magnetic selection (Miltenyi), and remaining CD4^+^ T cells were resuspended in cRPMI and 20 U/mL IL-2 before 1 × 10^6^ cells were plated per well of a 24-well plate for additional 19 days. Depletion of Vδ2 T cells was confirmed by flow cytometry showing a mean purity of 99% CD4^+^ T cells. On day 8, PHA-activated CD4^+^ T cells from HIV-seronegative donors were added to cultures to propagate outgrowth virus. Part of the media was replenished every 2–3 days, and supernatant was harvested on day 15 and 19 for HIV_p24_ ELISA quantification (R&D Systems). Results are reported as the number of positive wells compared between conditions.

### Statistics.

Statistical analyses were performed in GraphPad Prism or with the R programming language. Mann-Whitney *U* test was used to test for differences between ART-suppressed PWH and PWOH. Wilcoxon matched-pairs signed-rank test was used for paired analyses of phenotypes before and after expansion. Correlations between Vδ2 T cell phenotypes and participant clinical characteristics were performed using the Spearman’s ranked correlation test.

### Study approval.

Written informed consent was obtained from PWH at both the George Washington University Medical Faculty Associates and University of North Carolina, Chapel Hill, through IRB-approved protocols. Samples from PWOH purchased through the Gulf Coast Regional Blood Center were acquired without any associated personal identifiable information.

### Data availability.

Data values for all supporting figures within this study are available in the Supporting Data Values file.

## Author contributions

BTM designed, conducted, and analyzed experiments as well as wrote and edited the manuscript. NSS designed and supervised the study as well as wrote and edited the manuscript. M Sanz and AC conducted experiments. KL and M Siegel provided clinical support and samples for the study. All authors have approved the manuscript.

## Funding support

This work is the result of NIH funding, in whole or in part, and is subject to the NIH Public Access Policy. Through acceptance of this federal funding, the NIH has been given a right to make the work publicly available in PubMed Central.

- National Institute of Allergy and Infectious Diseases (NIAID) of the NIH under award no. R01-AI259057 and R21-AI157864 to NSS.
- Martin Delaney Collaboratory CARE (UM1 AI126619).
- District of Columbia Center for AIDS Research under an NIH funded program (P30AI117970).

## Supplementary Material

Supplemental data

Supporting data values

## Figures and Tables

**Table 1:**
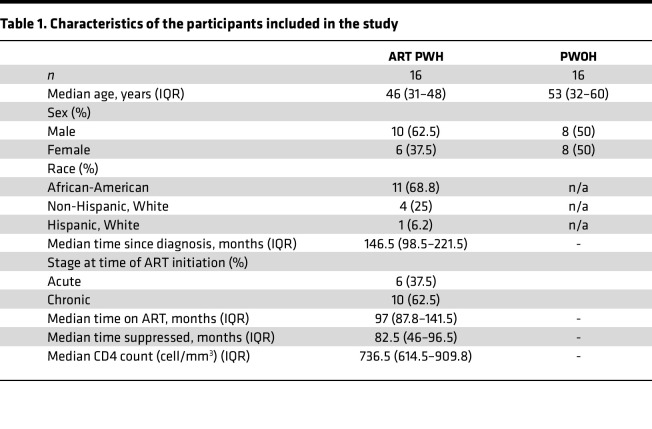
Characteristics of the participants included in the study

## Version 1 02/10/2026

In-Press Preview

## Version 2 03/23/2026

Electronic publication

## References

[1] Palmer S (2008). Low-level viremia persists for at least 7 years in patients on suppressive antiretroviral therapy. Proc Natl Acad Sci U S A.

[2] Barton K (2016). HIV-1 reservoirs during suppressive therapy. Trends Microbiol.

[3] Veenhuis RT (2023). Monocyte-derived macrophages contain persistent latent HIV reservoirs. Nat Microbiol.

[4] Ganor Y (2019). HIV-1 reservoirs in urethral macrophages of patients under suppressive antiretroviral therapy. Nat Microbiol.

[5] Pitman MC (2018). Barriers and strategies to achieve a cure for HIV. Lancet HIV.

[6] Archin NM (2012). Administration of vorinostat disrupts HIV-1 latency in patients on antiretroviral therapy. Nature.

[7] Sogaard OS (2015). The depsipeptide romidepsin reverses HIV-1 latency in vivo. PLoS Pathog.

[8] Borducchi EN (2016). Ad26/MVA therapeutic vaccination with TLR7 stimulation in SIV-infected rhesus monkeys. Nature.

[9] Sung JA (2018). HIV-specific, ex vivo expanded T cell therapy: feasibility, safety, and efficacy in ART-suppressed HIV-infected individuals. Mol Ther.

[10] Deng K (2015). Broad CTL response is required to clear latent HIV-1 due to dominance of escape mutations. Nature.

[11] Yamamoto T (2011). Surface expression patterns of negative regulatory molecules identify determinants of virus-specific CD8^+^ T-cell exhaustion in HIV infection. Blood.

[12] Connick E (2007). CTL fail to accumulate at sites of HIV-1 replication in lymphoid tissue. J Immunol.

[13] Collins KL (1998). HIV-1 Nef protein protects infected primary cells against killing by cytotoxic T lymphocytes. Nature.

[14] Huang SH (2018). Latent HIV reservoirs exhibit inherent resistance to elimination by CD8^+^ T cells. J Clin Invest.

[15] Clayton KL (2018). Resistance of HIV-infected macrophages to CD8^+^ T lymphocyte-mediated killing drives activation of the immune system. Nat Immunol.

[16] Clayton KL (2021). HIV-infected macrophages resist efficient NK cell–mediated killing while preserving inflammatory cytokine responses. Cell Host Microbe.

[17] Yukl SA (2013). The distribution of HIV DNA and RNA in cell subsets differs in gut and blood of HIV-positive patients on ART: implications for viral persistence. J Infect Dis.

[18] Trillo-Pazos G (2003). Detection of HIV-1 DNA in microglia/macrophages, astrocytes and neurons isolated from brain tissue with HIV-1 encephalitis by laser capture microdissection. Brain Pathol.

[19] Vantourout P, Hayday A (2013). Six-of-the-best: unique contributions of γδ T cells to immunology. Nat Rev Immunol.

[20] Sanz M (2023). Deep characterization of human γδ T cell subsets defines shared and lineage-specific traits. Front Immunol.

[21] Tanaka Y (1995). Natural and synthetic non-peptide antigens recognized by human gamma delta T cells. Nature.

[22] Wang H (2011). Indirect stimulation of human Vγ2Vδ2 T cells through alterations in isoprenoid metabolism. J Immunol.

[23] Kunzmann V (1999). Gamma/delta T-cell stimulation by pamidronate. N Engl J Med.

[24] Rincon-Orozco B (2005). Activation of V gamma 9V delta 2 T cells by NKG2D. J Immunol.

[25] Tokuyama H (2008). V gamma 9 V delta 2 T cell cytotoxicity against tumor cells is enhanced by monoclonal antibody drugs--rituximab and trastuzumab. Int J Cancer.

[26] Ryan PL (2016). Heterogeneous yet stable Vδ2(+) T-cell profiles define distinct cytotoxic effector potentials in healthy human individuals. Proc Natl Acad Sci U S A.

[27] Clohosey ML (2020). Comparable Vδ2 cell functional characteristics in virally suppressed people living with HIV and uninfected individuals. Cells.

[28] Li Z (2011). IFN-γ enhances HOS and U2OS cell lines susceptibility to γδ T cell-mediated killing through the Fas/Fas ligand pathway. Int Immunopharmacol.

[29] Dokouhaki P (2013). NKG2D regulates production of soluble TRAIL by ex vivo expanded human γδ T cells. Eur J Immunol.

[30] Xu Y (2021). Allogeneic Vγ9Vδ2 T-cell immunotherapy exhibits promising clinical safety and prolongs the survival of patients with late-stage lung or liver cancer. Cell Mol Immunol.

[31] Sanz M (2022). Human Vδ2 T cells and their versatility for immunotherapeutic approaches. Cells.

[32] Wallace M (1996). Gamma delta T lymphocyte responses to HIV. Clin Exp Immunol.

[33] Hudspeth K (2012). Engagement of NKp30 on Vδ1 T cells induces the production of CCL3, CCL4, and CCL5 and suppresses HIV-1 replication. Blood.

[34] Fausther-Bovendo H (2008). NKG2C is a major triggering receptor involved in the V[delta]1 T cell-mediated cytotoxicity against HIV-infected CD4 T cells. AIDS.

[35] Soriano-Sarabia N (2015). Peripheral Vγ9Vδ2 T cells are a novel reservoir of latent HIV infection. PLoS Pathog.

[36] Mann BT (2025). Dual role of circulating and mucosal Vδ1 T cells in the control of and contribution to persistent HIV-1 infection. Nat Commun.

[37] Garrido C (2018). γδ T cells: an immunotherapeutic approach for HIV cure strategies. JCI Insight.

[38] James KS (2020). Measuring the contribution of γδ T cells to the persistent HIV reservoir. AIDS.

[39] Garrido C (2018). Interlukin-15 stimulated natural killer cells clear HIV-1 infected cells following latency reversal ex vivo. J Virol.

[40] Li H, Pauza CD (2011). HIV envelope-mediated, CCR5/α4β7-dependent killing of CD4-negative γδ T cells which are lost during progression to AIDS. Blood.

[41] Field KR (2024). γδ T cells mediate robust anti-HIV functions during antiretroviral therapy regardless of immune checkpoint expression. Clin Transl Immunology.

[42] Alexander AA (2008). Isopentenyl pyrophosphate-activated CD56^+^ {gamma}{delta} T lymphocytes display potent antitumor activity toward human squamous cell carcinoma. Clin Cancer Res.

[43] Urban EM (2009). Control of CD56 expression and tumor cell cytotoxicity in human Vgamma2Vdelta2 T cells. BMC Immunol.

[44] Davis ZB (2017). CD155 on HIV-infected cells is not modulated by HIV-1 Vpu and Nef but synergizes with NKG2D ligands to trigger NK cell lysis of autologous primary HIV-infected cells. AIDS Res Hum Retroviruses.

[45] Richard J (2010). HIV-1 Vpr up-regulates expression of ligands for the activating NKG2D receptor and promotes NK cell–mediated killing. Blood.

[46] Cairo C (2010). Impact of age, gender, and race on circulating γδ T cells. Hum Immunol.

[47] Poonia B, Pauza CD (2012). Gamma delta T cells from HIV^+^ donors can be expanded in vitro by zoledronate/interleukin-2 to become cytotoxic effectors for antibody-dependent cellular cytotoxicity. Cytotherapy.

[48] Busca A (2009). Anti-apoptotic genes in the survival of monocytic cells during infection. Curr Genomics.

[49] Jenkins MR (2015). Failed CTL/NK cell killing and cytokine hypersecretion are directly linked through prolonged synapse time. J Exp Med.

[50] Prager I (2019). NK cells switch from granzyme B to death receptor-mediated cytotoxicity during serial killing. J Exp Med.

[51] Qin G (2009). Phosphoantigen-expanded human gammadelta T cells display potent cytotoxicity against monocyte-derived macrophages infected with human and avian influenza viruses. J Infect Dis.

[52] Tu W (2011). The aminobisphosphonate pamidronate controls influenza pathogenesis by expanding a gammadelta T cell population in humanized mice. J Exp Med.

[53] Garrido C (2016). HIV latency-reversing agents have diverse effects on natural killer cell function. Front Immunol.

[54] Cocchi F (1995). Identification of RANTES, MIP-1 alpha, and MIP-1 beta as the major HIV-suppressive factors produced by CD8^+^ T cells. Science.

[55] Chitrakar A (2022). HIV latency in myeloid cells: challenges for a cure. Pathogens.

[56] Desimio MG (2017). The histone deacetylase inhibitor SAHA simultaneously reactivates HIV-1 from latency and up-regulates NKG2D ligands sensitizing for natural killer cell cytotoxicity. Virology.

[57] Sanz M (2023). Aminobisphosphonates reactivate the latent reservoir in people living with HIV-1. Front Immunol.

[58] Mann BT (2020). Boosting the immune system for HIV cure: A γδ T cell perspective. Front Cell Infect Microbiol.

[59] Alnaggar M (2019). Allogenic Vγ9Vδ2 T cell as new potential immunotherapy drug for solid tumor: a case study for cholangiocarcinoma. J Immunother Cancer.

