# Supplementary material for: Ex vivo–expanded allogeneic Vδ2 T cells specifically reduce reservoirs of HIV-1 following latency reversal
Source: JCI Insight. 2026 Feb 10;11(6):e198185. doi: 10.1172/jci.insight.198185 (PMC13043098; doi:10.1172/jci.insight.198185)
Supplement: Supplemental data [file jciinsight-11-198185-s221.pdf]

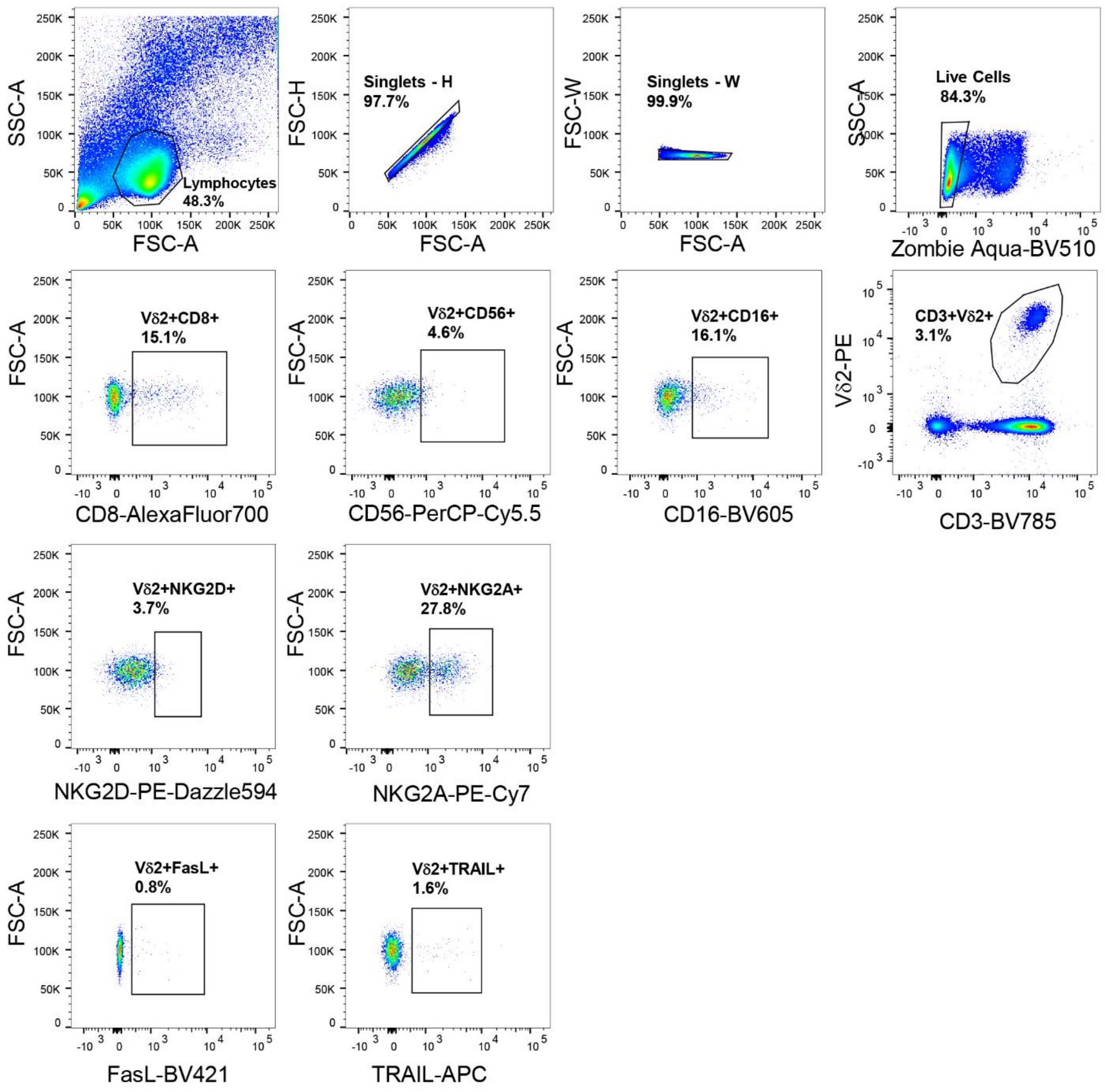

Supplemental Figure 1. Strategy for Vδ2 T cell phenotyping. Representative plots of cytotoxic markers measured used in phenotypic comparison of Vδ2 T cells from ART-suppressed PWH and PWOH.

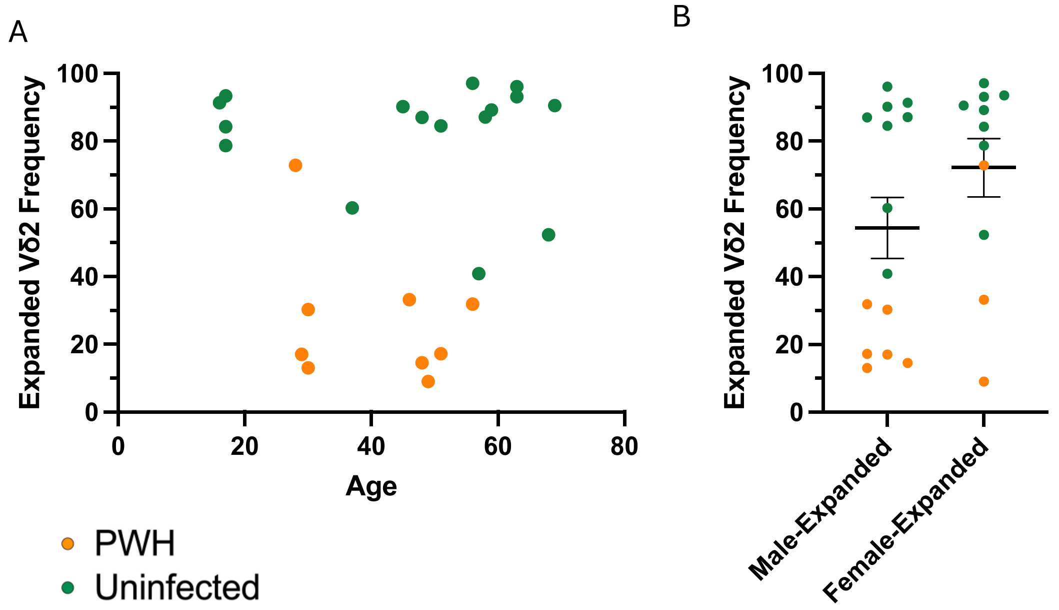

Supplemental Figure 2. Associations between participant characteristics and Vδ2 T cell expansion. (A) Lack of correlation between the frequency of expanded Vδ2 T cells in ART-suppressed PWH (orange) or PWOH (green). (B) Expanded Vδ2 T cell frequencies compared by biological sex. Spearman's ranked correlation test (A). Mann Whitney U-test (B).
